# Puberty in the African

**Published:** 1846-02

**Authors:** 


*Puberty in the African.*—An important law question, says the New York Sun of Thursday last, came before the Court of Common Pleas of Franklin county Ohio, in the case of Joseph Williams, a colored boy under 14 years of age, charged with an attempt to outrage the person of Althear S. McDougal, a child of five years of age. The charge was proven. It was contended for the defence that the prisoner, or a boy under 14 years of age, could not be punished for this offence, according to the English decisions. The prosecution held that these were inapplicable to the present case, and medical testimony was given to prove that persons of African descent arrive at the age of puberty earlier than Europeans. This decided the question, and the jury returned a verdict of guilty. He was sentenced to the Penitentiary for three years. His counsel intend to carry the case to the Supreme Court, says the same paper. It would be a matter of peculiar gratification here, to know the source of the medical testimony which so essentially influenced the court. If the discovery has actually been made that individuals of African descent sooner arrive at puberty than the descendants of other races of men, it is altogether a new fact in physiology—and the law of development was first promulgated, we believe, in the precincts of the Ohio tribunal. A physician is expected to state what he knows to be fact, and, if a court insists, he is justified also in advancing an opinion; but to stand up before a jury and positively declare that the descendants of Africans arrive at puberty earlier than the Caucasians or Mongolians, is assuming high ground, and what we think is not susceptible of proof.—*Boston Med. and Surg. Jour.*

